# Biomarkers selection and mathematical modeling in biological age estimation

**DOI:** 10.1038/s41514-023-00110-8

**Published:** 2023-07-01

**Authors:** Solim Essomandan Clémence Bafei, Chong Shen

**Affiliations:** Department of Epidemiology, Center for Global Health, School of Public Health, Nanjing Medical University, Nanjing, 211166 Jiangsu China

**Keywords:** Biomarkers

## Abstract

Biological age (BA) is important for clinical monitoring and preventing aging-related disorders and disabilities. Clinical and/or cellular biomarkers are measured and integrated in years using mathematical models to display an individual’s BA. To date, there is not yet a single or set of biomarker(s) and technique(s) that is validated as providing the BA that reflects the best real aging status of individuals. Herein, a comprehensive overview of aging biomarkers is provided and the potential of genetic variations as proxy indicators of the aging state is highlighted. A comprehensive overview of BA estimation methods is also provided as well as a discussion of their performances, advantages, limitations, and potential approaches to overcome these limitations.

## Introduction

Biological aging is a process or set of processes that cause the viability of organs to deteriorate over time^1^, while chronological aging refers to the passage of time. Biological age (BA), often used to measure the progress of biological aging, can indicate an aging individual’s life expectancy and health status^2^. Chronological age (CA) counts individuals’ years lived, not their functional level, life experience, or vulnerability to aging-related diseases and disabilities. Consequently, people with the same CA might age differently^3^, more likely due to variations in their fundamental biological aging mechanisms^4^. BA is affected by various factors such as heredity, physical fitness, and external environmental stressors^5^.

BA predicts mortality and the occurrence of various age-associated disorders, such as cardiovascular diseases (CVD), diabetes, cancers, and Alzheimer’s disease^4,6,7^. BA is useful for clinical and community surveillance and evaluating interventions to delay or prevent aging-related disease and disability^8^. BA estimations methods measure aging biomarkers and integrate these values into years using the evolving mathematical models^9^. To date, there is not yet standardized or widely accepted aging biomarkers and mathematical model for quantifying BA^10^.

Methods proposed to quantify BA in units of years include multiple linear regression (MLR)^11^, principal components analysis (PCA)^12^, Hochschild method (HocM)^13^, Klemera-Doubal method (KDM)^2,9^, machine learning (ML) algorithms^14^, and epigenetic age calculators^4,15–18^. Other methods, including homeostatic dysregulation (HD)^19^, allostatic load (AL)^20^, and polygenic risk scores (PRS)^21^ algorithms have evolved to explore biological aging. In this review, we aim to overview BA estimation processes covering the biomarkers selection and the choice of mathematical models to calculate BA. The performance of the different mathematical models in predicting age-related adverse health outcomes is compared, noting their benefits, drawbacks, and potential solutions to overcome these limitations.

## Aging biomarkers

An aging biomarker is a physiological measure used alone or combined with other variables to detect, diagnose, or forecast the functional competence or function loss of any biological component of a live organism in the absence of illness^22^. The American Federation of Aging Research (AFAR) recommends the following to select aging biomarkers^23^: (1) a biomarker should predict the extent of aging and lifespan better than CA; (2) it should control the primary mechanism that underlies aging, not be the result of illnesses; (3) it can be tested multiple times without harming anyone; (4) it can be effective in humans and experimental animals. In other words, biomarkers for BA estimations must predict the best lifetime than CA. Nevertheless, biomarkers that strictly fulfill AFAR guidelines are not likely to exist^24^.

Nakamura and Miyao^25^ also proposed and tested four other statistical approaches for aging biomarkers selection. According to their standards, (i) an aging biomarker should correlate significantly with CA cross-sectionally; (ii) the longitudinal change of an aging biomarker with CA should concord with cross-sectional correlation; (iii) there should be a significant steadiness of individual variability in the measures; (iv) the part of change caused by aging should be proportional to the variation in lifespan among the concerned species.

Generally, researchers select two main types of aging biomarkers: those based on specific cellular level modification (cellular biomarkers) and patient-level combining data from multiple clinical tests (clinical biomarkers), according to their biological role in the aging process. Typical methods of BA estimation as suggested by Nakamura and Miyao^25^ standard (i) correlate^11,26,27^ and/or regress^28,29^ candidates’ biomarkers on CA to select the significant ones according to established cut-off-points of their effect sizes to determine aging biomarkers. However, in the Levine method, biomarkers are selected based on their relationship with death, while in HocM, biomarkers are selected based on their association with life and death factors^4,13^.

### Cellular biomarkers

#### Genomic biomarkers

BA can reflect the life expectancy of an individual^30^. About a quarter of the variance in human longevity is due to genetic factors^31^. Telomeres are protective chromosomal ends of repeated DNA sequences bound by particular nucleoproteins and are lengthy at birth and diminish with each mitosis as age increases^32^ Telomere shortening is theorized to facilitate the physiological mechanism of aging^33^, longevity^34^, and death^35^.

Genome-wide association studies (GWAS) including Catalog lists 55 studies that revealed 676 genetic variants associated with aging, aging characteristics, lifespan, and longevity^36^. The variants of apolipoprotein (APO)E, and Forkhead box O3 A(FOXO3A) have replicated almost constantly in various populations^37,38^. APOE is a pleiotropic gene that has many functions including packaging and transporting low-density lipoprotein cholesterol^39^. Its polymorphisms are determined by three alleles: ε2, ε3, and ε4 defined by combinations of genotypes of the single nucleotide polymorphisms (SNPs) rs7412 and rs429358. APOE-ɛ2 is associated with increased odds of longevity while ɛ3 and ɛ4 are associated with shorter life expectancy^37,38^. Long-lived individuals often have a lower frequency of ɛ4, and a higher frequency of ɛ2 or a higher concentration of plasmatic APOE^40^ whereas careers of APOE-ɛ4 alleles often have abnormal lipid levels^41^ and are more at risk of Alzheimer’s disease, diabetes, and CVD^42^. Those diseases might accelerate biological aging and increase the likelihood of death which could explain the detrimental effect of APOE-ɛ4 on longevity.

There are about 40 noncoding SNPs in FOXO3A that have been repeatedly linked to longevity in humans^43^. FOXO3A is a component of the nutrient-sensing pathway connected to insulin and insulin growth factor (IGF)-1 regulates the cell response to oxidative stress and nutrition availability. It functions as a transcription factor on several homeostatic genes in response to diminished insulin or IGF-1 signaling^44^. The variants may boost FOXO3A to battle oxidative stress by strengthening its links with upstream and downstream molecular partners. In animal models, changes that affect these signals can delay the aging process since this pathway controls many facets of cell homeostasis, including cell survival and proliferation^44^. Another example of genetic variation associated with longevity is provided in the Supplementary information file.

##### Polygenic risk score as a predictor of longevity

One method of summarizing findings from GWAS for the prediction of complex variables, like longevity, is through polygenic risk scores (PRS). PRS are the weighted scores of independent variations that represent the genetic risk to develop a phenotypic characteristic. PRS for longevity can serve as a proxy indicator of biological aging as higher PRS for longevity might forecast a slower biological aging process and are associated with an increased likelihood of longevity^45^ whereas high PRS of life-threatening diseases are often associated with decreased odds of longevity^46^. High PRS for longevity is associated with an increased likelihood of healthy cognition aging and longer survival^47^ and is negatively associated with metabolic syndrome^48^ and brain aging process^49^. All these strengthen the idea that genetic variation contributing to lifespan might regulate the biological aging process.

#### Epigenetic biomarkers

Epigenetics is rapidly being recognized as an important fragment of aging and longevity^50^. Epigenetic modifications are theorized to facilitate the physiological mechanism of aging and are used as biomarkers to quantify BA^33,51^. Biomarkers based on DNA methylation (DNAm) which is an epigenetic modification characterized by a chemical change of genomic nucleotide bases^52^ met the various elusive criteria for a cellular biomarker of aging: they apply to all sources of DNA and across the CA spectrum^53^. Researchers apply ML algorithms to measure DNAm modification in multiple tissues, which enables the generation of highly accurate age estimators called epigenetic clocks. Numerous epigenetic clocks have emerged in recent years; the most popular ones are Hannum, Horvath, Levine, and Lu clocks, and genes that are associated with these four clocks’ age acceleration (AA) have been identified. These genes include PIK3CB a gene related to human longevity, CISD2 which has an important role in lifespan regulation^54^, TET2 involved in many aging/regenerative phenotypes^55^, and IBA57 a protein-coding gene that is linked to several disorders of mitochondrial malfunction^56^. The association of at least one epigenetic clock with aging and/or longevity-related genes suggests that epigenetic clocks might be valuable biomarkers of biological aging.

#### Transcriptional biomarkers

The expression of many genes exhibits age-related changes during growth and development^57^ and studies that have constructed transcriptomic age which is a BA from transcriptomic sources using these genes have reported relatively good results (mean absolute error (MAE) = 4.7 and 7.8 years)^57,58^. A previous study by Harries et al.^59^ showed that the molecular pathways mostly involved in the processing and maturation of messenger RNA transcripts were strongly related to increasing CA, and identified a group of six transcripts to distinguish successfully young and old people. The transcriptomic age correlated strongly with CA but not to the same extent as epigenetic clocks age in two studies^57,60^. Although the transcriptomic age was not a significant predictor of mortality in one study^60^, it was significantly related to smoking, waist-to-hip ratio, and systolic blood pressure in another study^57^. Furthermore, subjects with accelerated transcriptomic age had increased levels of total cholesterol and blood pressure^57^ whereas subjects with younger transcriptomic age displayed younger biochemical indices such as IL-6, blood urea, and serum albumin than their counterparts with older BA^61^. All these highlight the relevance of transcriptomic biomarkers in reflecting the biological aging state.

### Clinical biomarkers

Individual deteriorations in system integrity are theorized to be caused by molecular variations in aging, such as parameters that combine information from multiple clinical tests are the most used aging biomarkers to quantify BA^33^. Those parameters can be biochemical, physical, mental, and functional parameters that continuously vary with age^62^. The potential of metabolites and proteins as biomarkers for BA estimation is provided in the Supplementary information file. Table 1 presents some parameters used to calculate BA across studies.

**Table 1:** Parameters used to calculate BA across studies.

Clinical biomarkers	Groups	Parameters
	Musculoskeletal	Height^6^, waist circumference^6^, height and thigh circumference^28^, gait-handgrip strength^28^, knee extension strength^28^, timed up and go^28^, gait speed^28^, chair rise^28^, hands bones^62^
	Respiratory	Forced expiratory volume^25,28^
	Renal	Estimated glomerular filtration rate^6,28^, Albumin^26,63^, creatinine^26,63^, Urea nitrogen^6,26,28^, uric acid^63^
	Cardiovascular	Systolic blood pressure^6,25,63^, diastolic blood pressure^28^, cholesterol^6,26,63^, triglycerides^6,26^, high-density lipoprotein cholesterol^6^, Low-density lipoprotein cholesterol^26^
	Hepatic	Aspartate aminotransferase^6^, gamma-glutamyl transpeptidase^6^, alkaline phosphatase^6,26,63^, bilirubin
	Red blood cells	Hemoglobin^6,28^, hematocrit^26^, glycated hemoglobin^63^
	White blood cells and immune	Immunoglobulin M^26^, β2-microglobulin^26^, white blood cell count^63^, C-reactive protein^63^
Cellular biomarkers	Genome	Telomere length^34,64^, (rs8176719, rs687621, rs643434, and rs505922)^65^, (APOE-ɛ2, APOE-ɛ3, and APOE-ɛ4)^38,66^, (rs2069837 and rs2440012)^67^, rs2802292, (rs2764264 and rs13217795)
	Epigenome	Hannum clocks^15^, Horvath clocks^16^, Levine clocks^4^, and Lu clocks^17^
	Transcriptome	(LRRN3, CD27, GRAP, CCR6, VAMP5, CD248, D248, ABLIM1, LRRN3, CD27, GZMH, FAM102A, NELL2, SERPINE2, LEF1, CCR7 etc…)^57,59,61^

## BA estimation methods

### Multiple linear regression (MLR)

The MLR approach is widely used in small and large-scale studies^26,63^. Using MLR, BA is assumed to be equal to the predicted CA of an individual and based on the relationship between the measured CA and several biomarkers^11^. Durbina et al.^64^ provided an upgrade of MLR because of the distorted estimation of BA at the regression edges (called systematic error) with the equation below:

1 $${\rm{BAc}} = {\rm{BA}} + z$$

2 $$z = \left( {{\rm{CA}} - {\rm{Mean}}_{{\rm{CA}}}} \right) \times \left( {1 - b} \right)$$

BA’s simple linear regression coefficient on CA is *b*; BAc is the resulting BA after correction or improvement. MLR is easy to perform and provides BA estimates that correlate significantly with CA among healthy individuals (*r* > 0.80)^29,63^ and predicts mortality among adults^7^. Nevertheless, MLR is criticized because each biomarker’s weight effect depends on the strength of its link with CA. MLR’s systematic error persists despite its correction approaches; there is also a risk of multicollinearity^13^, which can be overcome by excluding highly intercorrelated biomarkers in the same model. Also, studies comparing mathematical models reported MLR as not being the best for BA estimation because of the low power of its BA estimates to predict adverse health events compared to other models^5,7,9,10,28^.

### Principal component analysis (PCA) method

PCA selects the least possible physiological parameters to quantify BA^12^ and is widely used to estimate BA^6,10,12,65^. With PCA, BA estimates are built in five phases: correlation analysis, constancy analysis, redundancy analysis, PCA, and final equation construction^65^. The last step, which consists of the equation construction, is built using the following equation^66^:

3 $${\rm{BAS}} = \frac{{a \times \left( {X_1 - {\rm{Mean}}{}_1} \right)}}{{{\rm{SD}}_1}} + \frac{{b \times \left( {X_2 - {\rm{Mean}}_2} \right)}}{{{\rm{SD}}_2}} + ..... + \frac{{m \times \left( {X_m - {\rm{Mean}}_m} \right)}}{{{\rm{SD}}_m}}$$

BAS, Mean_*m*_, SD_*m*_, *m*, *X*_*m*_ are BA score, the mean, standard deviation, coefficient obtained by PCA, and the values of biomarker *m*, respectively. It is suggested to transform BAS to BA because the unit of BAS is not in a year using the following equation^66^:

4 $${\rm{BA}} = \left( {{\rm{BAS}} \times {\rm{SD}}_{{\rm{CA}}}} \right) + {\rm{Mean}}_{{\rm{CA}}}$$

To avoid systematic error, BA is corrected using the following equation:

5 $${\rm{BAc}} = {\rm{BA}} + z\quad \quad \quad \quad \quad \quad \quad z = \left( 2 \right)$$

Increasing evidence shows that the BA estimates resulting from PCA can serve as an illness prediction tool to screen vulnerable populations and monitor the aging degree^67^. As an example, BA estimates resulting from PCA predicted metabolic syndrome^68^ and reduced survival likelihood^69^ in different studies.

PCA is simple to understand and perform compared to ML, HocM, and KDM^67^. PCA outclassed only MLR in predicting frail individuals^28^ and has shown a good linear fit with CA with a mean regression slope of 0.96^10^ and 1^5^, respectively. However, MLR and PCA share the same correction method by using *z*-scores to correct the BAS, which has been found inappropriate because of the persistence of the distortion of the BA estimates at regression edges^13^. PCA limitations also include essential parameters exclusion^70^, contributing to a loss of information^71^.

### Hochschild’s method (HocM)

HocM consists of assessing biomarkers based on their influence on lifespan and death. Hochschild^72^ chose physiological parameters according to their sensitivity to CA found in previous studies, “quality of life criterion”, their ability to adapt to the devised instrument to quantify biomarkers, and their correlation with mortality. To consider the selected parameters as biomarkers of aging, they were correlated with the composite validation variable (CVV) based on eight death risk factors grouped in smoking status, diet, exercise level, lifespan, and education level^72^. Each risk factor was assigned a particular weight and the risk factors scores were standardized and combined into the mean validation variable (MVV) following the equation below:

6 $${{{\mathrm{MVV}}}} = \frac{{\mathop {\sum}\nolimits_{i = 1}^n {W_i{\rm{SF}}_i} }}{{\mathop {\sum}\nolimits_{i = 1}^n {W_i} }}$$

where *W*_*i*_ and SF_*i*_ are the weight and the standardized scores of factor *i* and *n* is the total number of risk factors. CVV is obtained by standardizing MVV^72^. HocM was the first to use the reverse regression technic in the BA estimation process. In HocM, *n* linear regression equations of biomarkers on CA replace multiple linear regression of CA on biomarkers.

7 $$Y_i = d_i + e_i{{{\mathrm{CA}}}}\left( { + f_i\,K} \right)$$

*i* = 1 ~ *n*, *K* is height and the term in brackets is added only when *i* designates a height-dependent biomarker such as forced expiratory volume and forced vital capacity. Stepwise mathematical procedures are performed to get the standardized BA (SBA), an intermediate BA product. The final phase of HocM is to convert the SBA to BA in years units. This is accomplished by employing a concept similar to Stanford-IQ Binet’s index, which requires selecting an arbitrary mean and standard deviation^72^. There is a need for a cautious exploration of which subject’s death risk factors will be included in CVV and a large sample size to apply HocM to a newly developed system^9^. HocM is a challenging multiple-stepwise approach that is not widely used for BA estimation.

### Klemera and Doubal method (KDM)

KDM is a graphing method developed by Klemera and Doubal^2^ to compute BA. KDM uses the reverse regression technique proposed in HocM for the BA estimation process in which CA is used as a standard biomarker, not an outcome to be predicted, enabling a substantial improvement in BA estimates’ precision. The following equation was formulated using many mathematical steps^2^:

8 $${\rm{BA}} = \frac{{\mathop {\sum}\nolimits_{i = 1}^n {\left( {x_i - g_i} \right)\frac{{h_i}}{{s_i^2}} + \frac{E}{{s_D^2}}} }}{{\mathop {\sum}\nolimits_{i = 1}^n {\left( {\frac{{h_i}}{{s_i}}} \right)^2 + \frac{1}{{s_D^2}}} }}$$

*E* stand for CA, *x*_*i*_ is the value of biomarker *i*, *g*_*i*_, *h*_*i*_, *s*_*i*_ is the regression slope, the intercept, and the root mean squared error (RMSE) from a regression of *i* on CA, and $s_D^2$ is equal to the variance in CA explained by the *n* biomarkers. BA equations are built using computer-generated simulations; this advanced concept makes KDM an optimal method for BA estimation^2^. Studies that have compared BA estimates resulting from KDM and other models in mortality prediction found that KDM was an excellent method to compute BA than the previous algorithms^5,7,10,28^ but recent algorithms such as HD and ML outperformed KDM in predicting various health outcomes including mortality^20,73^.

### Machine learning (ML) approaches for BA estimation

The ML approach, a generic artificial intelligence technique in which a computer learns data using a specially developed algorithm or without one is rapidly emerging and encourages biological aging and longevity research^74^. ML can be supervised, unsupervised, and semi-supervised. Table 2 represents the ML algorithms used to compute BA in some studies. ML algorithms are broad and have provided relatively reliable BA estimates with prediction accuracy of 0.87–4.76 years and *R*^2^ of 0.25–0.94^73,75^. ML algorithms are useful for big data and estimating BA from non-conventional parameters, including radiograph^76^ and magnetic resonance imaging results^77^. Nevertheless, they are subjected to some limitations: there is not yet a specific algorithm in ML specially dedicated to BA estimation; also, many ML algorithms have a “black box” effect that prevents a systematic understanding of the relationships between features and labels, as well as how they are estimated^78^. In this scenario, traditional statistical tools and comprehension of medical or biological topics are helpful. Also, there is a risk of overfitting with many biomarkers in the same model^79^. Recently Yang et al.^80^ suggested a composite ML-BA based on the stacking technique with a simple meta-model to avoid overfitting and improved the performance of the BA estimates in predicting different health outcomes^80^. A brief description of the stacking method is provided in the Supplementary information file.

**Table 2:** ML algorithms used to compute BA in some studies.

Studies	ML algorithms
Putin et al.^80^	Gradient boosting machine (GBM), random forest (RF), decision trees (DT), k-nearest neighbors (KNN), elastic net regression (ENR), Support vector machine (SVM)
Rahman et al.^81^	Deep neural network (DNN), convolutional neural network (CNN), convolutional long short-term memory
Egorova et al.^82^	RF, SVM
Sagers et al.^83^	RF
Galibourg et al.^84^	AdaBoost, Bayesian ridge regression (BRR), DT, KNN, multi-layer perceptron (MLP), polynomial regression (POLYREG), RF, SVM, voting regressor
Wang et al.^78^	ENR, SVM, partial least squares, extreme gradient boosting (Xgboost)
Bae et al.^85^	POLYREG, SVM, Xgboost, DNN, RF
Yang et al.^86^	Xgboost, Catboost, Adaboost, Extra Trees, DNN, CNN, Light gradient boosting machine
Pyrkov et al.^14^	CNN
Levine et al.^4^, Hannum et al.^15^, Horvath^16^, Belsky et al.^18^	ENR

### Epigenetic age calculators

#### First-generation epigenetic clocks

Hannum, Horvath, Levine, and Lu clocks are the most popular clocks that estimate epigenetic age, which is BA from DNA sources. Hannum clock is the first-reported trained and tested clock based on blood-derived DNA. Hannum’s epigenetic age model was built using ENR analysis. It encompasses 71 CpGs chosen from the Illumina 450k array, which powerfully captures CA variations, partly driven by age-related shifts in blood cell composition^15^.

The Horvath clock was built using several tissues, including the blood data from Hannum as a likely “pan-tissue” master clock of CA, and focused on catching common changes independent of tissue type. The Horvath epigenetic age model was built using ENR analysis. It encompassed 353 CpGs on the previous Illumina 27k array^16^. Horvath’s epigenetic age can be estimated using an online age calculator (http://dnamage.genetics.ucla.edu/home). A study reported Horvath’s epigenetic age performing Hannum in correlation with CA^81^ whereas another study reported Hannum’s epigenetic age as having the highest correlation with CA^82^.

#### Second-generation epigenetic clocks

##### DNAm PhenoAge

Levine et al.^4^ built a BA model using biomarkers from the whole blood sample of individuals in NHANES III. The hazard of death due to aging was regressed on 44 clinical parameters and CA in a Cox penalized regression model for choosing ten variables to include in their epigenetic age model called phenotypic age. The ten parameters were encompassed in a parametric proportional hazards model based on the Gompertz distribution. Based on that model, individuals’ 10 years of death hazard were estimated. Subsequently, the death score was transformed into years^4^. Using ENR analysis, the phenotypic age estimates were regressed on DNAm data. The penalization parameter has been selected to lessen the cross-validated mean-square error rate, resulting in 513 CpGs. The PhenoAge is strongly correlated with CA (*r* = 0.94) and outclasses earlier epigenetic clocks in predicting all-cause death, malignancies, healthspan, Alzheimer’s disease^4^, and breast cancer^83^.

##### DNAm GrimAge

DNAm GrimAge is a composite biomarker based on seven DNAm substitutes and an estimator of the self-reported number of cigarettes smoked yearly based on a DNAm developed by Lu et al.^17^. The GrimAge was constructed in two steps. The first was to define surrogate DNAm biomarkers for physiological stressors and risk variables and the second step consisted of integrating these biomarkers to create DNAm GrimAge, a single composite biomarker of longevity that is expressed in years using elastic net Cox regression^17^. The authors further developed GrimAge 2 following the same steps as in the previous GrimAge with the difference that they added two plasmatic biomarkers: CRP and hemoglobin AIC which were log-transformed^84^. The GrimAge 2 estimation approach is implemented in an online software (https://dnamage.clockfoundation.org/). The GrimAges outperformed the previously reviewed clocks and clinical biomarkers related to BA in predicting aging-related ailments and all-cause death^60,85,86^.

#### Third-generation epigenetic clocks

##### Dunedin pace of aging (PA) methylation

Belsky et al.^18^ developed DNAm-related PA using the data from Dunedin cohort participants who had 18 biomarkers measured in three waves (age 26, 32, and 38) and who had DNAm data available at age 38. They applied ENR using the PA (please see the Supplementary Information file for PA estimation method description) as the criterion variable and all methylation probes found on both the Illumina 450k and EPIC arrays as predictors. A total of 46 CpG sites was associated with the longitudinal PA and the resulting Dunedin PA methylation was more strongly associated with aging-related ailments than PA^18^.

##### Dunedin Pace

Dunedin Pace measure differed from Dunedin PA methylation in four points: (1) PA analysis included data from 20 years of follow-up; (2) PA analysis included four-time points of measurement; (3) Dunedin Pace modeling excluded CpG sites for which probes having low reliability in blood datasets; and (4) the algorithm used to implement Dunedin Pace includes a normalization step that allows Dunedin Pace values for individual samples to be compared to the Dunedin^87^. The final Dunedin Pace algorithm included 173 CPG sites. It has a high reproducibility with the intraclass correlation (ICC) greater than 0.87 across databases which enables it to be used as a reliable measurement tool for aging intervention trials^87^.

##### Advantages and limitations of epigenetic clocks and age estimators

The epigenetic age estimates have yielded impressively robust correlations with CA with minimal variance and great consistency across multiple age groups and studies’ populations^88^. However, epigenetic age calculators have some limitations: instead of limiting the set to biologically important pathways, epigenetic age calculators take hundreds of CpGs as input. As a result, they are not as informative to identify intervention targets or molecular pathways. In addition, epigenetic clocks often have low reliability with the ICC in the poor to moderate range^89,90^, resulting in up to 3 to 9 years difference between technical replicates and providing false positive or negative associations^90^. To overcome this limitation, Higgins-Chen et al.^90^ proposed and trained epigenetic clocks using principal components instead of individual CpGs which contributed significantly to clock reliability improvement with ICCs 0.990–0.998^90^.

## Other measures of biological aging

### Homeostatic dysregulation (HD) algorithm

HD offers an alternative to exploring biological aging and has been used for that purpose across studies^8,27,91,92^. Subjects’ HD shows the variation of their physiology from a reference in good health based on a biomarker Mahalanobis distance (MD)^92^. MD is the multidimensional distance of an individual’s biomarkers values from a single reference point^93^. A higher HD score reflects a higher BA, risk of illness, incapacity, and death, while a smaller HD score reflects the opposite. HD does not assume any unidirectionality of aging biomarkers relationship with CA and does not incorporate CA into its calculation^8^.

Before calculating HD, a reference population should be defined; in some cases, the reference is the mean of all values, however, in other cases is theory-driven, such as the mean of values in a young, healthy population representing the maximum of reproductive fitness. Then, biomarker values from the reference population should be standardized and used to calculate a biomarker variance-covariance matrix. The biomarker raw mean and SD, and the standardized-biomarker variance-covariance matrix should be applied within the Mahalanobis distance equation to obtain the HD algorithm^93^:

9 $${\rm{HD}}_1 = \sqrt {( {\mathop{w}\limits^{\rightharpoonup} - \mathop{y}\limits^{\rightharpoonup}} )^T \times S^{ - 1} \times ( {\mathop{w}\limits^{\rightharpoonup} - \mathop{y}\limits^{\rightharpoonup}} )}$$

$\mathop{w}\limits^{\rightharpoonup}$ is a vector of biomarkers values of a subject in the study; $\mathop{y}\limits^{\rightharpoonup}$ is a vector of biomarker means in the reference sample, and *S* is the reference sample variance-covariance matrix. If none of the parameters are correlated, the HD score is obtained by scaling each biomarker by its variance and adding the deviance squared for each biomarker^93^.

10 $${\rm{HD}}_2 = \sqrt {\mathop {\sum}\nolimits_{i = 1}^n {\frac{{\left( {w_i - y_i} \right)^2}}{{{\rm{SD}}^2\left( {x_i} \right)}}} }$$

SD^2^(*x*_*i*_) is the variance of biomarker *i*, and *n* is the number of biomarkers. HD estimates have predicted morbidity, mortality, and functional deficit^8^. A study reported that HD estimates outperformed KDM in predicting mortality^94^ whereas another study showed that KDM outperformed HD^33^. The HD algorithm has limitations: the statistical distance depends on detecting a normal biological state, often calculated as the population average for all parameters, a reasonable imperfect estimate. Finding the ideal centroid is not easy; therefore, the dysregulation score could be systematically biased^91^.

### Allostatic load algorithms

The AL is wear and tear on the body and brain caused by persistent overactivity or inactivity of biological systems that are often involved in response to environmental stimulus^95^. While AL has been often used to explore how physiological stress influences different outcomes, Hasting et al.^20^ demonstrated that AL and primary measures of biological aging such as KDM and HD share similarities in their fundamental concepts: Similar to biochemistry parameters of systemic BA, AL assessment incorporates systemic biomarkers to form a measure of cumulative dysregulation; furthermore, AL theory is theoretically relevant for BA processes because psychosocial stress can accelerate the progression of several aging signs. The authors further estimated AL scores using three methods as well as calculated two measures of biological aging using KDM and HD. The three different AL estimates had a moderate correlation with KDM-BA (*r* = 0.34–0.39) and a moderate to stronger correlation with HD estimates (*r* = 0.46–0.68), suggesting that the three estimates might share some similarities in reflecting the aging process. Subjects with higher AL and HD scores and KDM-BA performed worse on physical agility tests and reported being in worse physical and mental health. Through these, AL might be considered a proxy measure of biological aging. There are over 30 methods of AL estimation which were reviewed in a recent literature review^96^.

### Polygenic risk score algorithms

A polygenic risk score is a weighted score of independent variants that quantifies the genetic likelihood of developing a certain trait. PRS is typically calculated as follows:

11 $${\rm{PRS}}_i = \mathop {\sum}\nolimits_j^M {{\rm{ES}}_j \ast D_{ji}}$$

*M* is the number of variants in the score, the effect size (ES) of the variants *j*, and dosage (*D*_*ji*_) is the number of copies of SNP *j* in the genotype of individual *i*. The ES is often gotten from a discovery GWAS^21^. Other variations of the classical method of PRS^46,97^ have been proposed and software was developed to facilitate PRS calculation^98^. The step-by-step construction of polygenic scores has been reviewed in a recent study^99^.

## Summary of biological aging estimation algorithms

Although each method has its advantages and limitations, all the BA estimates from the reviewed BA estimation methods have relatively good prediction power of various health outcomes than CA in many studies. Table 3 represents the summary of the BA estimation algorithms.

**Table 3:** Summary of the BA estimation algorithms.

Methods	Merits	Limitations
MLR	MLR is easy to understand and perform	-Each biomarker’s weight effect depends on the power of its relationship with CA
	BA estimates from MLR are relatively reliable and have good prediction power	
		-Distortion of BA estimates at the regression edges (systematic error)
		-Risk of multicollinearity
PCA	PCA is easy to understand and perform	-The exclusion of important parameters
	It solves the major limitation of MLR	-Loss of information due to parameters dimensionality reduction in PCA
	PCA-BA estimates are effective in illness prediction to screen vulnerable populations and monitor the aging degree	-Inadequacy of the proposed correction
HocM	HocM avoids distortion of BA estimates at the regression edges (systematic error)	There is limited information on HocM because that approach is not widely used
	HocM considers CA as an independent variable	HocM is difficult to be implemented in systems other than its author’s own
KDM	It considers CA as a standard biomarker	
	It solves the major limitations of MLR	
	BA estimates resulting from KDM are highly predictive of illnesses, impairments, and mortality	
ML	ML is useful for big data and non-usual parameters	There is limited evidence that ML methods are better than conventional methods
		Risk of overfitting
		There is not yet an explicit algorithm in ML mainly dedicated to BA estimation
Epigenetic age calculators	Epigenetic age calculators provide BA estimates that correlated highly with CA	-Epigenetic age calculators take hundreds of CpGs as input instead of limiting the set to biologically important pathways limiting their ability to identify intervention targets or molecular pathways
	Epigenetic age calculators use DNAm clocks as biomarkers	
		-DNAm clocks have low reproducibility
		-Risk of overfitting
	They are highly predictive of illnesses and mortality	
HD	HD does not assume the unidirectionality of aging biomarkers’ relationship	Finding the ideal centroid is not easy, making the dysregulation score biased systematically
	HD does not incorporate CA into its calculation	
	HD estimates are highly predictive of morbidity, mortality, and functional deficit	

## Discussion

Biomarkers selection is an integral part of the BA estimation process, and the standards for biomarkers selection vary depending on BA estimation methods and researchers. Furthermore, many BA estimation approaches presume a linear relationship between biomarkers and CA. A linear model presupposes a constant increase or decrease of the biomarkers’ values over time which might not accurately reflect the complex aging process taking place in the body. In reality, aging can occur at different rates for different individuals and in different parts of the body^100^ making the assumption of linearity difficult to establish. Additionally, many aging biomarkers such as telomere length or clinical parameters are influenced by a multitude of factors that are not necessarily linked directly to age, such as lifestyles, stress, and genetic predisposition. Thus, relying on a linear relationship between biomarkers and age may not accurately capture the true relationship between them. Furthermore, there is an increasing body of evidence showing a linear and non-linear variation of proteins, clinical biomarkers composites, and gene expression throughout the life course in healthy and general population^75,101,102^. Lehallier et al.^102^ reported a non-linear variation of more than two thousand plasmatic proteins across the lifespan in both healthy humans and mice and in human replication cohorts. The authors further pinpointed hundreds of proteins varying in waves throughout the lifespan and summing the number of differentially expressed proteins at each age revealed three peaks at ages 34, 60, and 78. Additionally, there was a statistically significant overlap between proteins altering in the three age groups but the majority of proteins altering in old age were not identified by the linear model^102^. These results highlight the inadequacy of linear modeling to capture the complexity of biological aging across the lifetime. Thus, relying only upon a linear association assumption of parameters and CA to select aging biomarkers might exclude potentially valid aging biomarkers that have a non-linear association with CA and result in inaccurate BA estimates. Therefore, both linear and non-linear associations of parameters and CA are worth exploring in the biomarker’s selection process.

Progress in technology and easy data accessibility through open-access databases have contributed to an increased number of studies exploring biological aging. To date, the National Institute on Aging has listed over 20 publicly available databases for aging-related research^103^, and their data have been extensively used in biological aging investigations. All these have not only enabled the identification of complex age-related patterns of potential aging biomarkers^75,102^ but have also raised some concerns about the data quality. There is a lack of standardized measurement methods making it difficult to compare and contrast results from different studies. For example, methods commonly used in large epidemiological studies for telomere length measurement are frequently associated with substantial measurement error^104^. As a result, even when results are consistent and of high quality, they may be difficult to compare between laboratories. A recent study dropped telomere length from its list of biomarkers to be used for biological aging exploration because of the yet-unresolved field-wide controversies about its measurement^105^; these highlight the need to develop standardized biomarkers measurement tools.

In this study, we have summarized current knowledge on aging biomarkers and BA estimation methods as well as discussed their performances in predicting numerous age-related diseases, their advantages, shortcomings, and potential solutions to overcome those shortcomings. The validity of the BA estimates highly depends on the choice of biomarkers and the method applied for its calculation. Previous studies have assessed the utility of different biomarkers and BA estimation methods have identified the best mathematical model based on the association between its BA estimates and CA and its capacity to predict various health outcomes compared to others. However, the constancy of the predictive ability of these BA estimates is inconclusive across multiple studies because the model that correlated the best with CA and/or predicts various outcomes in one study might fail in another study. Therefore, there is a need to clarify which parameter(s) and mathematical model might provide a trustworthy indicator of biological aging.

## Supplementary information

Supplementary information file
