# Supplementary material for: Biomarkers selection and mathematical modeling in biological age estimation
Source: NPJ Aging. 2023 Jul 1;9(1):13. doi: 10.1038/s41514-023-00110-8 (PMC10314900; doi:10.1038/s41514-023-00110-8)
Supplement: Supplementary file 1 — Supplementary information file [file 41514_2023_110_MOESM1_ESM.pdf]

SUPPLEMENTARY INFORMATION

Genomic biomarkers (continued)

Recently, Ni et al.[1] discovered and confirmed the presence of four variants in ABO genes that are related to longevity in a Chinese cohort. The comparison of genotype and metabolic phenotypes revealed that long-lived persons with rs8176719, rs687621, rs643434, and rs505922 were related to normal lipid concentration and BMI. The authors proposed two routes involving vWF/ADAMTS13 and inflammatory indicators that co-regulated lipid concentration via glycosylation and had mutual effects. As a result, persons with longevity-associated variants have a better cardiovascular profile, which may minimize the risk of aging-related illnesses and preserve healthy physical conditions, resulting in a longer life expectancy[1]. All these results highlight the potential predisposition for accelerated or slower biological aging and could serve as proxy indicators of biological aging.

Proteomic biomarkers

Proteins have a direct impact on physiological processes, therefore they might provide more useful information about aging and age-related disorders[2]. Several studies have identified proteins in human tissues and bodily fluids that appear to change consistently with age[3–8] and developed proteomic age predictors of BA termed proteomic age that was highly correlated with CA ($r = 0.84$-0.97)[5,6,9]. The proteomic age is associated with morbidities and mortality but not strongly as CA in one study[4] but outperformed CA and frailty index in predicting all-cause mortality among older adults in another study[10]. Studies have shown that subjects with younger proteomic age were more likely to have better scores in physical and cognitive tests[6] and proteomic age correlated more strongly with CA ($r = 0.84$) than transcriptomic and metabolomic age, and telomere length

(r = 0.72, 0.70, and 0.30, respectively)[9]. All these results show that proteins might be relevant biomarkers for BA estimation. Johnson et al.[3] compared the performance of proteomic age obtained using a set of 83 and 23 proteins reported to change with CA. The 23 proteins-based proteomic age had a correlation coefficient of 0.66 and a mean delta age of 8.54 years whereas the 83 proteins-based proteomic age had a correlation coefficient of 0.87, and the mean delta age of 5.5 years in the test datasets[3], suggesting that using many proteins might provide more accurate BA estimates than using few proteins.

Metabolomic biomarkers

Metabolites are small molecules that are substrates, intermediates, and products of metabolic events occurring in the body. They are essential for energy production, and signal and information transmission about the body's condition and ongoing processes[11]. Evidence has shown that metabolite levels change with aging even when no disease is present among children and adults[12,13]. Studies using metabolites alone to compute metabolomic age which is another type of BA from metabolic sources are not broad compared to other types of biomarkers. Studies that have estimated metabolomic age reported that metabolomic age correlated significantly with CA (r = [0.58 to 0.86][9,14] and subjects with accelerated metabolomic age had an increased risk of CVD, lower functionalities, increased BMI, high alcohol, diabetes, depression, and death[14,15]. However, there is no association between accelerated epigenetic and metabolomic aging suggesting that they capture separate aspects of biological aging[14].

The stacking method

Stacking is an ensemble ML technique combining the learned categories of models into a single model that consists of base models and meta-models. Stacking uses a meta-model to balance the output of the base model and forecast rather than selecting a model from many models for generalization or simple averaging. The stacking allows the improvement of the prediction performance by combining data of prediction output from numerous various models to make the new dataset cover more potential features. Furthermore, a simple meta-model such as linear regression or a generalized additive model can decrease the likelihood of model overfitting and have a better generalization ability resulting in the meta-model having comparable fitting effects in the training and testing sets in the second layer. However, an extremely complex meta-model, on the other hand, will result in overfitting[16]. The authors did not evaluate the generalizability of the stacking method using a simple meta-model in an external dataset; therefore, further studies are required for its validation.

Pace of aging

The pace of aging (PA) quantifies an individual's rate of biological decline over time and was developed for longitudinal data with repeated biomarkers measurement. PA was originally calculated with eighteen biomarkers obtained from a birth cohort with no age-related diseases from which biomarkers were measured at ages 26, 32, and 36 years. Its estimation process includes three stages. The first stage consists of transforming all the biomarkers into z-scores based on the reference age distribution separately for males and females. The second stage consists of applying mixed-effects growth models to compute each study participant's slope for each of the biomarkers. The model takes the equation below:

$$D_{it} = F_0 + F_1 CA_{it} + h_{oi} + h_{1i} CA_{it} + E_{it} \quad (1)$$

D_{it} stands for biomarkers of participant *i* at time *t*, F_0 and F_1 are the fixed intercept and slopes estimated for the cohort and h_{oi} and h_{1i} are the random intercept and slope estimated for participants *i*. In the third step, each study participant's PA was calculated as the sum of age-dependent annual changes in biomarkers z-scores.

$$PA_i = \sum_{D=1}^{n} h_{1iD} \quad (2)$$

h_{1iD} is the slope of biomarker *D* for Participant *i*[17]. The PA can be scaled to show biological change over time according to a population norm. In the original study, the cohort means PA was set as a reference value that equates to the biological change expected during a single chronological year. The quantification of PA enables a demonstration of the difference in aging-related decline among subjects with the same CA might reflect their biological aging process. Participants from the same CA with faster PA exhibit higher cognitive impairments, indicators of advanced brain aging, reduced sensory-motor abilities, and older physical appearance[17,18].

REFERENCES

1. Ni, X. *et al.* Identification and replication of novel genetic variants of ABO gene to reduce the incidence of diseases and promote longevity by modulating lipid homeostasis. *Aging (Albany. NY).* **13**, 24655–24674 (2021).

2. Moaddel, R. *et al.* Proteomics in aging research: A roadmap to clinical, translational research. *Aging Cell* **20**, e13325 (2021).

3. Johnson, A. A., Shokhirev, M. N., Wyss-Coray, T. & Lehallier, B. Systematic review and analysis of human proteomics aging studies unveils a novel proteomic aging clock and identifies key processes that change with age. *Ageing Res. Rev.* **60**, 101070 (2020).

4. Tanaka, T. *et al.* Plasma proteomic biomarker signature of age predicts health and life span. *Elife* **9**, (2020).

5. Tanaka, T. *et al.* Plasma proteomic signature of age in healthy humans. *Aging Cell* **17**, e12799 (2018).

6. Lehallier, B. *et al.* Undulating changes in human plasma proteome profiles across the lifespan. *Nat. Med.* **25**, 1843–1850 (2019).

7. Ignjatovic, V. *et al.* Age-related differences in plasma proteins: how plasma proteins change from neonates to adults. *PLoS One* **6**, e17213 (2011).

8. Menni, C. *et al.* Circulating Proteomic Signatures of Chronological Age. *J. Gerontol. A. Biol. Sci. Med. Sci.* **70**, 809–816 (2015).

9. Jansen, R. *et al.* An integrative study of five biological clocks in somatic and mental health. *Elife* **10**, (2021).

10. Sathyan, S. *et al.* Plasma proteomic profile of age, health span, and all-cause mortality in older adults. *Aging Cell* **19**, e13250 (2020).

11. Balashova, E. E., Maslov, D. L., Trifonova, O. P., Lokhov, P. G. & Archakov, A. I. Metabolome Profiling in Aging Studies. *Biology (Basel).* **11**, (2022).

12. Gu, H. *et al.* 1H NMR metabolomics study of age profiling in children. *NMR Biomed.* **22**, 826–833 (2009).

13. Johnson, L. C. *et al.* The plasma metabolome as a predictor of biological aging in humans. *GeroScience* **41**, 895–906 (2019).

14. Robinson, O. *et al.* Determinants of accelerated metabolomic and epigenetic aging in a UK cohort. *Aging Cell* **19**, e13149 (2020).

15. van den Akker, E. B. *et al.* Metabolic Age Based on the BBMRI-NL (1)H-NMR Metabolomics Repository as Biomarker of Age-related Disease. *Circ. Genomic Precis. Med.* **13**, 541–547 (2020).

16. Yang, Q. *et al.* A machine learning-based data mining in medical examination data: a biological features-based biological age prediction model. *BMC Bioinformatics* **23**, 411 (2022).

17. Belsky, D. W. *et al.* Quantification of biological aging in young adults. *Proc. Natl. Acad. Sci. U. S. A.* **112**, E4104-10 (2015).

18. Elliott, M. L. *et al.* Disparities in the pace of biological aging among midlife adults of the same chronological age have implications for future frailty risk and policy. *Nat. aging* **1**, 295–308 (2021).
